# Role of KRAS-LCS6 polymorphism in advanced NSCLC patients treated with erlotinib or docetaxel in second line treatment (TAILOR)

**DOI:** 10.1038/srep16331

**Published:** 2015-11-17

**Authors:** Monica Ganzinelli, Eliana Rulli, Elisa Caiola, Marina Chiara Garassino, Massimo Broggini, Elena Copreni, Sheila Piva, Flavia Longo, Roberto Labianca, Claudia Bareggi, Maria Agnese Fabbri, Olga Martelli, Daniele Fagnani, Maria Cristina Locatelli, Alessandro Bertolini, Giuseppe Valmadre, Ida Pavese, Anna Calcagno, Maria Giuseppa Sarobba, Mirko Marabese

**Affiliations:** 1 Oncology Department, Fondazione IRCCS Istituto Nazionale dei Tumori, Milan, Italy; 2 Oncology Department, IRCCS - Istituto di Ricerche Farmacologiche Mario Negri, Italy; 3 Oncology Department, Ospedale Fatebenefratelli e Oftalmico, Milan, Italy; 4 Medical Oncology Department, Policlinico Umberto I Rome, Italy; 5 Oncology Department, Papa Giovanni XXIII Hospital, Bergamo, Italy; 6 Medical Oncology, Fondazione IRCCS Ca’ Granda - Ospedale Maggiore Policlinico, Milano, Italy; 7 Medical Oncology Department, Ospedale Belcolle, Viterbo, Italy; 8 Medical Oncology Department, San Giovanni e Addolorata Hospital, Rome, Italy; 9 Oncology Department, Azienda Ospedaliera Desio e Vimercate, Vimercate, Italy; 10 San Carlo Borromeo Hospital, Milano, Italy; 11 Valtellina e Valchiavenna Hospital, Sondrio, Italy; 12 Valtellina e Valchiavenna Hospital, Sondalo, Italy; 13 San Pietro Hospital, Roma, Italy; 14 Legnano Hospital, Legnano, Italy; 15 Azienda Ospedaliera Universitaria, Sassari, Italy

## Abstract

MicroRNAs were described to target mRNA and regulate the transcription of genes involved in processes de-regulated in tumorigenesis, such as proliferation, differentiation and survival. In particular, the miRNA *let-7* has been suggested to regulate the expression of the *KRAS* gene, a common mutated gene in non-small cell lung cancer (NSCLC), through a *let-7* complementary site (LCS) in 3′UTR of KRAS mRNA. We have reported the analysis performed on the role of the polymorphism located in the KRAS-LCS (rs61764370) which is involved in the disruption of the *let-7* complementary site in NSCLC patients enrolled within the TAILOR trial, a randomised trial comparing erlotinib versus docetaxel in second line treatment. In our cohort of patients, KRAS-LCS6 polymorphism did not have any impact on both overall survival (OS) and progression free survival (PFS) and was not associated with any patient’s baseline characteristics included in the study. Overall, patients had a better prognosis when treated with docetaxel instead of erlotinib for both OS and PFS. Considering KRAS-LCS6 status, the TG/GG patients had a benefit from docetaxel treatment (**HR**_**(docetaxel vs erlotinib)**_ = 0.35, 95% CI 0.15–0.79, p = 0.011) compared with the TT patients (**HR**_**(docetaxel vs erlotinib)**_ = 0.72, 95% CI 0.52–1.01, p = 0.056) in terms of PFS.

Lung cancer is the first cause of cancer-related death in Western countries^1^. This malignancy is strongly associated with environmental factors and smoking^2^. The prognosis of patients with Non-Small Cell Lung Cancer (NSCLC) is very poor with a percentage of survivors that is lower than 15% for all stages and lower than 5% in metastatic disease^3^.

*KRAS* is one of the most frequently mutated genes in NSCLC, in fact its mutations are present in approximately 20% of this type of tumour. *KRAS* belongs to the *ras* family and it encodes a small G protein with intrinsic GTPase activity, which is necessary for protein inactivation, and to tune the downstream effectors involved in pathways such as proliferation and differentiation. Mutations in defined aminoacids determine the loss of intrinsic GTPase activity and the deregulation of downstream pathways^4^. In addition to mutations, KRAS activity can be altered through a lower protein expression promoted by miRNA binding to its messenger RNA. A polymorphic site in the 3′ untranslated region of *KRAS*, is able to eliminate the ability of miRNA let-7 to bind to the target. The single nucleotide polymorphism (SNP) (rs61764370), named KRAS let-7 complementary site (KRAS-LCS6), was described as the change of the T-allele to a G-allele. This modification was seen to increase the KRAS expression and to activate the downstream pathways. The KRAS-LCS6 variant is not very common and the G-allele frequency is about 7% in the European population^5^.

The KRAS-LCS6 was associated with higher cancer risk in triple-negative breast cancer^6^ and reduced survival in oral cancer patients^7^. On the contrary, the KRAS-LCS6 SNP was associated with a better outcome in early stage colorectal cancer, but this feature was lost in advanced stages of this disease^8^. In ovarian cancer the KRAS-LCS6 polymorphism was described to have the opposite role and also no function^9^,^10^,^11^. In lung cancer, the moderate smoker population harbouring the G-allele was shown to have an increased cancer risk^5^ but the presence of infrequent allele did not reduce the survival rate of patients^12^.

Since *KRAS* mutation demonstrated only a little impact on survival, as also reported in TAILOR trial results^13^, and KRAS activity can be regulated by microRNA, patients stratification based only on KRAS status could not be sufficient to evaluate the role of this biomarker.

Given that the prognostic and predictive role of KRAS-LCS6 polymorphism was not yet investigated in lung cancer, we planned an ancillary study to assess the value of KRAS-LCS6 polymorphism on outcomes within the TAILOR trial, a randomised trial comparing erlotinib versus docetaxel in second line NSCLC.

## Results

Between October 2007 and March 2012, 222 eligible patients were enrolled in the TAILOR trial. Among 222 randomised patients (110 to docetaxel and 112 to erlotinib), 218 were fully eligible for the main trial^14^. Of these, 145 (82.4%) had TT genotype in the KRAS-LCS6 locus, 30 (17.1%) harboured a TG variant whereas only one (0.5%) patient had GG polymorphism (hereafter included in the TG patients group). For the remaining 42 patients, we were not able to collect blood samples. The CONSORT diagram is illustrated in the Supplementary Figure S1. The minor allele prevalence was 10%, consistent with available data. The baseline characteristics of the patients included in the present study according to KRAS-LCS6 polymorphism are illustrated in Table 1.

For the TT population the median age at diagnosis was 66 years (interquartile range (IQR): 58.8–71.4 years) whereas it was 70 years (IQR: 60.9–73.3 years) for the TG/GG population. The TT group was predominantly stage IV (51.7%), had adenocarcinoma histology (69.0%), poorly differentiated grade (54.7%), a smoking habit (77.9%), ECOG-PS of 0 (47.6%) and a wild-type status of KRAS (77.9%). Similarly, the TG/GG patients were predominantly stage IV (54.8%), with adenocarcinoma histology (74.2%), poorly differentiated grade (65.0%), smoking habit (71.0%), ECOG-PS of 0 (58.1%) and a wild-type status of KRAS (64.5%).

Although the polymorphism variants were not a stratification marker, the patients were well distributed between the two treatments performed in the main trial. In particular, 48.3% and 51.6% of TT and TG/GG patients respectively were treated with docetaxel. On the other hand, 51.7% of TT and 48.4% of TG/GG patients received erlotinib. None of the characteristics considered were associated with the different genotypes present in the polymorphic site.

### Survival outcomes

After a median follow-up of 33.0 months (IQR: 21.4–33.4), 170 patients progressed or died and 150 died.

**Figure 1:**
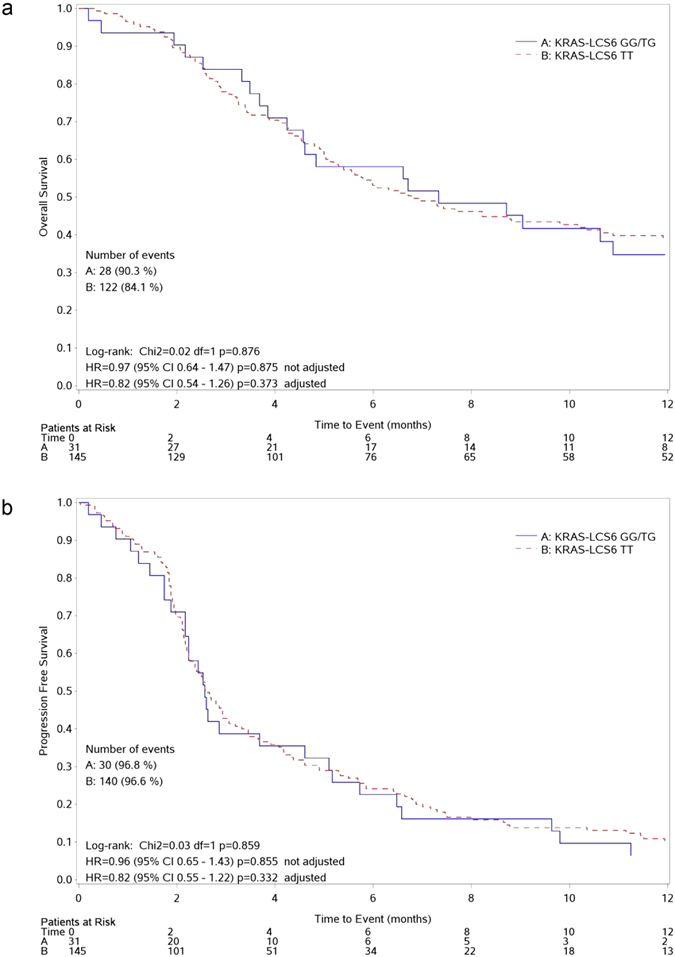
Kaplan-Meier curves for OS (**a**) and PFS (**b**) according to KRAS-LCS6 genotype.

The baseline characteristics associated with overall survival (OS) were: ECOG-PS (HR_(2 vs. 1 vs. 0)_ = 2.14, 95% CI 1.60–2.85, p < 0.0001) and sex (HR_(F vs M)_ = 0.68, 95% CI 0.47–0.97, p = 0.035). All risk estimate covariates are reported in Table 2. Median OS was 6.8 months (IQR 3.3–20.2 months) in the TT group and 7.3 months (IQR 3.7–15.3 months) in the TG/GG group (unadjusted HR_(TT vs TG/GG)_ = 0.97, 95% CI 0.64–1.47, p = 0.875; adjusted HR_(TT vs TG/GG)_ = 0.82, 95% CI 0.54–1.26, p = 0.373). Figure 1a shows the OS curves according to the KRAS-LCS6 polymorphism.

ECOG-PS (HR_(2 vs. 1 vs. 0)_ = 1.79, 95% CI 1.37–2.34, p < 0.0001) and treatment arm (HR_(docetaxel vs erlotinib)_ = 0.65, 95% CI 0.48-0.89, p = 0.007) were associated with progression free survival (PFS). All risk estimate covariates are reported in Table 3. Median PFS was the same for both groups: 2.6 months (IQR 1.9–5.9 months) in the TT group and 2.6 months (IQR 1.7–5.7 months) in the TG/GG group (unadjusted HR_(TT vs TG/GG)_ = 0.96, 95% CI 0.65–1.43, p = 0.855; adjusted HR_(TT vs TG/GG)_ = 0.82, 95% CI 0.55–1.22, p = 0.332). Figure 1b shows the PFS curves according to the KRAS-LCS6 polymorphism.

### Subgroup analyses

**Figure 2:**
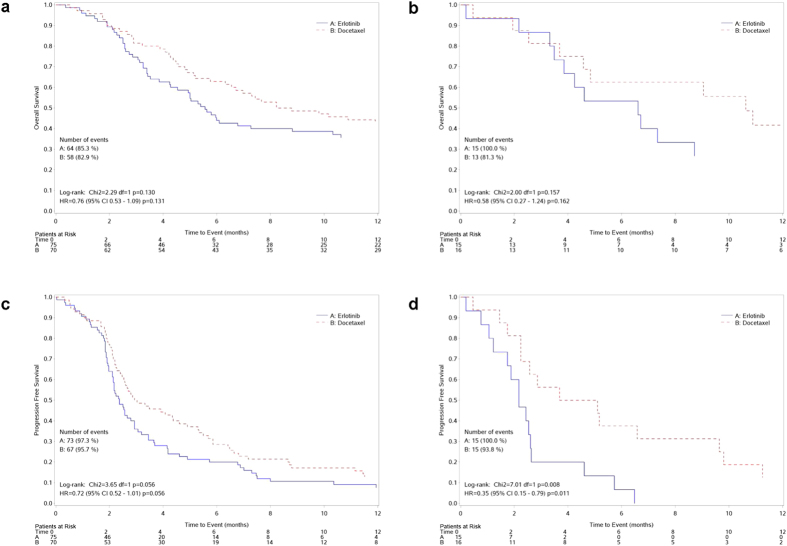
Kaplan-Meier curves reporting OS (upper panels) and PFS (lower panels) in TT (panels (**A,C**) and TG/GG (panels (**B,D**) patients according to treatment arm.

**Figure 3:**
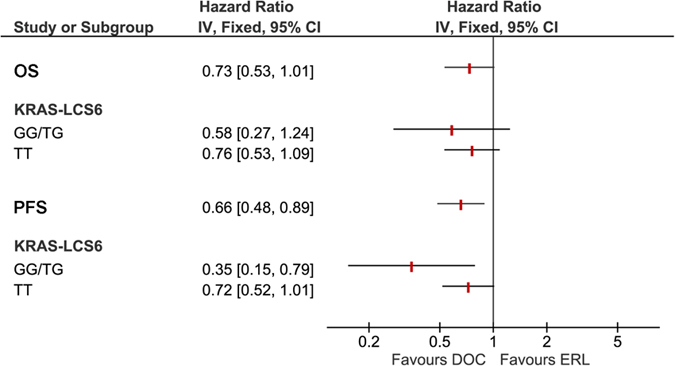
Forest Plots showing the predictive role of KRAS-LCS6 polymorphism.

For explorative purposes, we performed a subgroup analysis according to KRAS-LCS6 polymorphism status with the aim of investigating its predictive role on treatment efficacy. In patients with TT polymorphism, although not statistically significant, the risk of death was lower in the docetaxel compared to the erlotinib treated group (HR_(docetaxel vs erlotinib)_ = 0.76, 95% CI 0.53-1.09, p = 0.131). The same was observed for the TG/GG population (HR_(docetaxel vs erlotinib)_ = 0.58, 95% CI 0.27-1.24, p = 0.162). The test of interaction was not statistically significant (p = 0.618). The same was true for patients with TT polymorphism in terms of PFS. The risk of progression was lower in the docetaxel compared to the erlotinib treated group (HR_(docetaxel vs erlotinib)_ = 0.72, 95% CI 0.52-1.01, p = 0.056). On the other hand, we observed a much better PFS in response to docetaxel compared to erlotinib for the TG/GG population (HR_(docetaxel vs erlotinib)_ = 0.35, 95% CI 0.15-0.79, p = 0.011). Again, the test of interaction was not significant (p = 0.133). The curves reporting OS and PFS by treatment in TT and TG/GG patients are reported in Figure 2 while Figure 3 reports the Forest plot for the predictive role of KRAS-LCS6 polymorphism.

On the other hand, considering separately the different treatment arm we observed no difference in OS between the two polymorphisms both in docetaxel (HR_(TT vs TG/GG)_ = 1.01, 95% CI 0.55–1.86, p = 0.966) and in erlotinb arm (HR_(TT vs TG/GG)_ = 0.88, 95% CI 0.49–1.58, p = 0.676).

The same was observed in PFS both in docetaxel (HR_(TT vs TG/GG)_ = 1.18, 95% CI 0.67-2.07, p = 0.560) and in erlotinb arm (HR_(TT vs TG/GG)_ = 0.65, 95% CI 0.37-1.15, p = 0.140). The curves reporting OS and PFS by genotypes in the treatment arms are reported in the Supplementary Figure S2.

**Figure 4:**
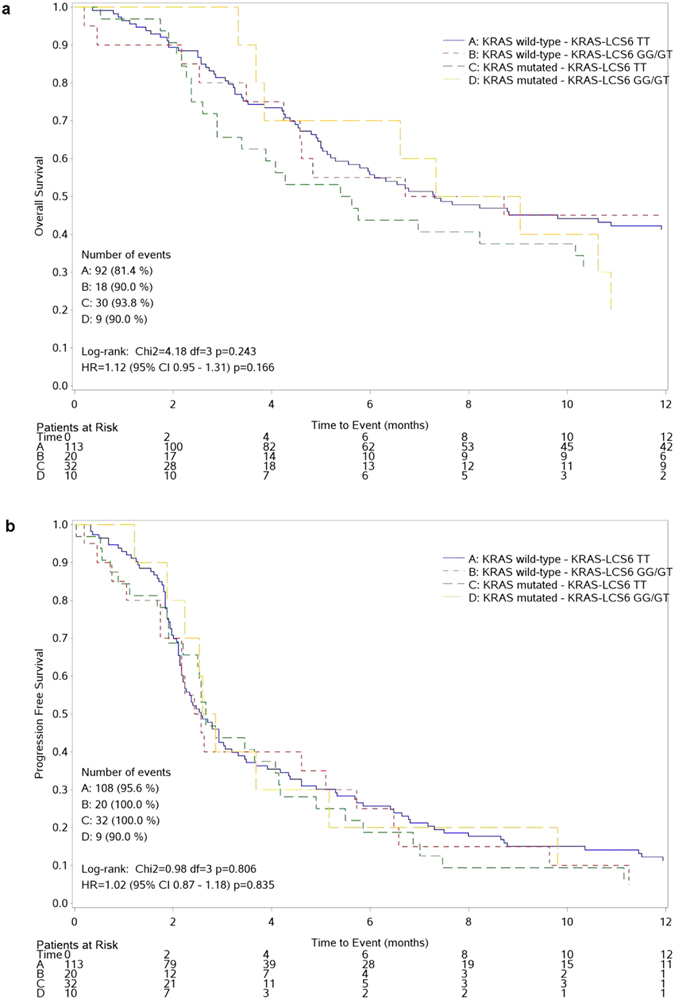
Kaplan-Meier curves reporting OS (upper panels) and PFS (lower panels) by KRAS status and genotypes.

We performed a second explorative analysis to address the role of KRAS-LCS6 polymorphism in the presence of either wild-type or mutated KRAS. In the presence of a wild-type KRAS both OS and PFS were almost equivalent when the two genotypes were compared (HR_(TT vs TG/GG)_ = 0.93, 95% CI 0.55–1.57, p = 0.792 for OS and HR_(TT vs TG/GG)_ = 0.88, 95% CI 0.54–1.41, p = 0.586 for PFS). When we considered a KRAS mutated background, the TG/GG genotypes seemed to indicate a protective trend in both OS and PFS although not statistically significant (HR_(TT vs TG/GG)_ = 1.29, 95% CI 0.61–2.74, p = 0.501 and HR_(TT vs TG/GG)_ = 1.27, 95% CI 0.60–2.67, p = 0.534 respectively). The test of interaction was not significant for both OS (p = 0.263) and PFS (p = 0.344). The curves reporting OS and PFS by KRAS status and genotypes are reported in Figure 4.

## Discussion

In the last two decades, many studies have been published analysing the prognostic and predictive roles of *KRAS* mutations in sustaining resistance to different types of treatment such as EGFR Tyrosine-Kinase Inhibitors (TKIs) and chemotherapy^15^,^16^,^17^. *KRAS* mutated patients were indicated to have a worse prognosis and resistance to treatment in different types of cancer but no clear conclusions have been stated for NSCLC^18^. The data were highly variable since extracted from retrospective studies, which either considered a very small number of patients or evaluated *KRAS* mutational status only in a subgroup of patients. Another possible reason could be the fact that, in addition to mutations and amplification, KRAS activity can be regulated by microRNA (miRNA), in particular miRNA let-7b^5^. For these reasons patients stratification based only on KRAS status could not be sufficient to evaluate the role of this biomarker. MicroRNAs let-7 were described as a family of miRNAs able to regulate the expression of some lung cancer oncogenes including *KRAS*^19^,^20^.

In the present work, we analysed the role of the genomic variant present in KRAS-LCS6 within a phase III clinical trial (TAILOR). The TAILOR trial was a non-profit multicentre, open label, randomised trial, conducted in 52 Italian hospitals, comparing erlotinib versus docetaxel in second line NSCLC^14^. Blood samples were collected with the aim of investigating any association between biomarkers and clinical/histopathological characteristics of the patients and the role of biomarkers possibly involved in the outcomes.

In our study, the genomic variant present in KRAS-LCS6 was not associated with any clinical or histopathological characteristics of the patients included in the study. Furthermore, as already reported by Nelson *et al.*^12^, our study confirms that the *KRAS* mutation prevalence was the same in both the genotype groups. We can support the Nelson hypothesis that occurs despite the up-regulation of KRAS expression, due to the G variant present in the let-7 binding site in the 3’UTR of KRAS, which did not result in any selective pressure for *KRAS* mutations.

Let-7 was originally identified in *Caenorhabditis elegans* as a regulator of developmental timing and cellular proliferation^21^ and, when ectopically expressed in cancer cell lines and xenograft models, miRNA let-7 was able to repress cellular proliferation^22^,^23^.

Let-7 expression levels were found to be reduced in NSCLC patients and this decrease has been associated with a worse clinical outcome^24^.

The same effect of let-7 levels reduction can also be obtained by the lack of miRNA binding site, as happens with the KRAS-LCS6 SNP, but we were not able to confirm the association between polymorphisms and poor prognosis given that, in our study, the TG/GG genotypes did not correlate with any outcome. We have no explanation for the lack of role for this polymorphism in our study. We cannot exclude that the expression levels of miRNA let-7 could be different among patients and nullify the impact of the different genotypes. It is also true that the marked role for KRAS-LCS6 polymorphism has usually been described in the case-control studies assessing cancer risk. In fact, an increased NSCLC risk was described as associated with the polymorphism and this was most evident among people who were light to moderate smokers^5^.

As reported in the main TAILOR trial and confirmed in this study, patients had a better prognosis in terms of PFS and OS when treated with docetaxel instead of erlotinib.

Because of the lack of the statistical power necessary to demonstrate a predictive effect of KRAS-LCS6, our study can only suggest that KRAS-LCS6 confers a different magnitude of the effect of docetaxel compared to erlotinb on both PFS and OS. In this view, considering KRAS-LCS6 status to stratify patients and perform explorative subgroup analysis, the TG/GG subgroup seemed to benefit more from docetaxel treatment when compared to erlotinib in terms of PFS.

The consideration on statistical power was true also for a second subgroup analysis considering the *KRAS* status to stratify patients. Patients in *KRAS* wild-type subgroup with TT genotype had slightly better outcomes whereas in the mutated *KRAS* population the contrary was observed.

In conclusion, the previous TAILOR results on the superiority of the chemotherapy in the absence of an identified target^14^ is once more confirmed in all subgroups analysed. Our data suggest that the KRAS-LCS6 polymorphism is not a critical prognostic factor but could identify a subgroup of patients (TG/GG) for which the use of a chemotherapy treatment seems to be extremely important.

## Methods

### Study design and patients

TAILOR was a non-profit multicentre, open label, randomised trial, funded by the Italian Regulatory Agency AIFA and conducted in 52 Italian hospitals, comparing erlotinib versus docetaxel in second line NSCLC. Details have been published previously^13^. Within the TAILOR trial we pre-planned a number of ancillary studies including the role of polymorphism on outcomes. Participating hospitals registered all consecutive patients with metastatic, recurrent or inoperable locally advanced NSCLC. Only those with both a *EGFR* and *KRAS* centrally determined status were included in the trial. All patients received a first line platinum-based chemotherapy in combination with either vinorelbine, gemcitabine or pemetrexed according to the physician’s decision. Combinations with taxanes and with anti-EGFR agents were not allowed. Patients with EGFR mutations were selectively treated with EGFR Tyrosine-Kinase Inhibitors (TKI) and were excluded from this analysis. All patients had an Eastern Cooperative Oncology Group (ECOG) Performance Status (PS) between 0 and 2 and were at least 18 years of age. Exclusion criteria included any evidence of serious co-morbidities that the investigator judged as a contraindication to the participation in the study, as well as pregnancy and breast-feeding. Research protocol was approved by the Ethics Committee of Ospedale Fatebenefratelli e Oftalmico, Milan (03 October 2007) and all patients who were eligible for participation provided written informed consent with all applicable governing regulations before undergoing any study procedure. All experiments were performed in accordance with the Declaration of Helsinki. The study was registered March 12, 2008 at ClinicalTrials.gov, number NCT00637910.

### Samples collection and genotyping

Blood specimens were collected in K_2_EDTA sample tubes and frozen at −80 °C. DNA was extracted from blood samples using Maxwell 16 DNA Purification Kit (Promega, Milan, Italy). The rs61764370 SNP was genotyped using a TaqMan SNP Genotyping assay (Applied Biosystems, Monza, Milan), based on Real Time PCR technique (ABI 7900, Applied Biosystems). The PCR was carried out in a 384-wells plate with a reaction volume of 5 μL containing genomic DNA (10 ng), 2× TaqMan Genotyping Master Mix (Applied Biosystems), 40× MGB probes and primers. Primers and probe sequences (MGB probes specifically designed for Allelic Discrimination) are property of Applied Biosystems. Thermal cycle conditions were 95 °C for 10 minutes and 40 cycles at 95 °C for 15 seconds and 60 °C for 1 minute. Completed PCR plates were analysed using the Allelic Discrimination Sequence Detection Software (Applied Biosystems).

### Statistical methods

Baseline covariate distributions were summarised using descriptive statistics (median and range for continuous variables; absolute and percentage frequencies for categorical variables); Wilcoxon-Mann-Whitney test for continuous covariates and Chi-square test for categorical covariates were used to detect statistical association. Progression Free Survival was defined as the time from the date of randomisation up to the date of first progression or death from any cause, whichever came first. Subjects who had not progressed or died while in the study were censored at the last disease assessment date. Overall survival was defined as the time from the date of randomisation up to the date of death from any cause. Subjects who did not die while in the study were censored at the last follow-up. Survival curves were estimated with the Kaplan-Meier method. Cox proportional hazards models were used for univariate and multivariate (adjusted for ECOG-PS and treatment arm) analysis to estimate the association between KRAS-LCS6 polymorphism and PFS and OS. Results were expressed as Hazard Ratios (HRs) and their 95% confidence intervals (95% CIs). Statistical analyses were carried out using SAS version 9.2 (SAS Institute, Cary, NC).

## Additional Information

**How to cite this article**: Ganzinelli, M. *et al.* Role of KRAS-LCS6 polymorphism in advanced NSCLC patients treated with erlotinib or docetaxel in second line treatment (TAILOR). *Sci. Rep.* **5**, 16331; doi: 10.1038/srep16331 (2015).

## Supplementary Material

Supplementary Information

## Figures and Tables

**Table 1:** Patient characteristics.

		TT		TG/GG		*P-value*
		*N*	%	*N*	%	
Patients		145	82.4	31	17.6	
Age	Median(quartile)	66.0 (58.8–71.4)		70.0 (60.9–73.3)		0.119
Sex	Male	97	66.9	23	74.2	0.430
	Female	48	33.1	8	25.8	
ECOG-PS	0	69	47.6	18	58.1	0.241
	1	66	45.5	12	38.7	
	2	10	6.9	1	3.2	
Smoking	Never	32	22.1	9	29.0	0.407
	Ex smokers/smokers	113	77.9	22	71.0	
Stage at diagnosis	I	14	9.7	2	6.5	0.852
	IIA	4	2.8	3	9.7	
	IIB	5	3.5	2	6.5	
	IIIA	25	17.2	4	12.9	
	IIIB	16	11.0	3	9.7	
	IIIB wet	6	4.1	0	0.0	
	IV	75	51.7	17	54.8	
Grading	G1	5	5.3	2	10.0	0.933
	G2	36	37.9	5	25.0	
	G3	52	54.7	13	65.0	
	Undifferentiated	2	2.1	0	0.0	
	unknown	50		11		
Histotype	Adenocarcinoma	100	69.0	23	74.2	0.640
	Squamous	34	23.5	7	22.6	
	Bronchoalveolar	2	1.4	1	3.2	
	Large cells	2	1.4	0	0.0	
	Other	7	4.9	0	0.0	
KRAS status	Wild type	113	77.9	20	66.7	0.190
	Mutated	32	22.1	10	33.3	
Treatment arm	Docetaxel	70	48.3	16	51.6	0.737
	Erlotinib	75	51.7	15	48.4	

**Table 2:** Prognostic evaluation of clinical and histopatological characteristics – Overall Survival.

	HR	Lower 95% HR	Upper 95% HR	*P-value*
Univariate				
KRAS-LCS6 (TT vs TG/GG)	0.97	0.64	1.47	0.875
Age at diagnosis	1.02	1.00	1.03	0.058
Treatment arm (docetaxel vs erlotinib)	0.73	0.53	1.01	0.060
Sex (F vs M)	0.68	0.47	0.97	0.035
Smoking (smoking and ex vs not smoking)	1.23	0.83	1.81	0.297
Tumour grade	1.19	0.86	1.64	0.292
Tumour stage (IIIBw/IV vs III vs I/II)	1.19	0.95	1.49	0.126
ECOG-PS (2 vs. 1 vs. 0)	2.14	1.60	2.85	<.0001
Histotype (squamous vs others)	1.15	0.78	1.70	0.467
*KRAS* (mut vs wt)	1.36	0.94	1.96	0.106
**Multivariate**				
KRAS-LCS6 (TT vs TG/GG)	0.82	0.54	1.26	0.373
Treatment arm (docetaxel vs erlotinib)	0.73	0.53	1.01	0.060
ECOG-PS (2 vs. 1 vs. 0)	2.17	1.62	2.91	<.0001

**Table 3:** Prognostic evaluation of clinical and histopatological characteristics – Progression Free Survival.

	HR	Lower 95% HR	Upper 95% HR	*P-value*
Univariate				
KRAS-LCS6 (TT vs TG/GG)	0.96	0.65	1.43	0.855
Age at diagnosis	1.01	0.99	10.2	0.267
Treatment arm (docetaxel vs erlotinib)	0.65	0.48	0.89	0.007
Sex (F vs M)	0.76	0.55	1.05	0.100
Smoking (smoking and ex vs not smoking)	1.32	0.92	1.89	0.129
Tumour grade	1.19	0.88	1.60	0.251
Tumour stage (IIIBw/IV vs III vs I/II)	1.16	0.94	1.42	0.172
ECOG-PS (2 vs. 1 vs. 0)	1.79	1.37	2.34	<.0001
Histotype (squamous vs others)	1.22	0.85	1.74	0.278
*KRAS* (mut vs wt)	1.04	0.73	1.48	0.822
**Multivariate**				
KRAS-LCS6 (TT vs TG/GG)	0.82	0.55	1.22	0.332
Treatment arm (docetaxel vs erlotinib)	0.65	0.48	0.89	0.007
ECOG-PS (2 vs. 1 vs. 0)	1.80	1.37	2.36	<.0001
