# Supplementary material for: Role of KRAS-LCS6 polymorphism in advanced NSCLC patients treated with erlotinib or docetaxel in second line treatment (TAILOR)
Source: Sci Rep. 2015 Nov 17;5:16331. doi: 10.1038/srep16331 (PMC4648064; doi:10.1038/srep16331)

Role of KRAS-LCS6 polymorphism in advanced NSCLC patients treated with erlotinib or docetaxel in second line treatment (TAILOR)

Monica Ganzinelli[1], Eliana Rulli[2], Elisa Caiola[2], Marina Chiara Garassino[1], Massimo Broggini[2], Elena Copreni[2], Sheila Piva[3], Flavia Longo[4], Roberto Labianca[5], Claudia Bareggi[6], Maria Agnese Fabbri[7], Olga Martelli[8], Daniele Fagnani[9], Maria Cristina Locatelli[10], Alessandro Bertolini[11], Giuseppe Valmadre[12], Ida Pavese[13], Anna Calcagno[14], Maria Giuseppa Sarobba[15] and Mirko Marabese[2*]

[1]Oncology Department, Fondazione IRCCS Istituto Nazionale dei Tumori, Milan, Italy;
[2]Oncology Department, IRCCS - Istituto di Ricerche Farmacologiche Mario Negri, Italy;
[3]Oncology Department, Ospedale Fatebenefratelli e Oftalmico, Milan, Italy;
[4]Medical Oncology Department, Policlinico Umberto I Rome, Italy;
[5]Oncology Department, Papa Giovanni XXIII Hospital, Bergamo, Italy;
[6]Medical Oncology, Fondazione IRCCS Ca' Granda - Ospedale Maggiore Policlinico, Milano, Italy
[7]Medical Oncology Department, Ospedale Belcolle, Viterbo, Italy.
[8]Medical Oncology Department, San Giovanni e Addolorata Hospital, Rome, Italy
[9]Oncology Department, Azienda Ospedaliera Desio e Vimercate, Vimercate, Italy
[10]San Carlo Borromeo Hospital, Milano, Italy
[11]Valtellina e Valchiavenna Hospital, Sondrio, Italy
[12]Valtellina e Valchiavenna Hospital, Sondalo, Italy
[13]San Pietro Hospital, Roma, Italy
[14]Legnano Hospital, Legnano, Italy
[15]Azienda Ospedaliera Universitaria, Sassari, Italy

Supplementary Figure S1: CONSORT

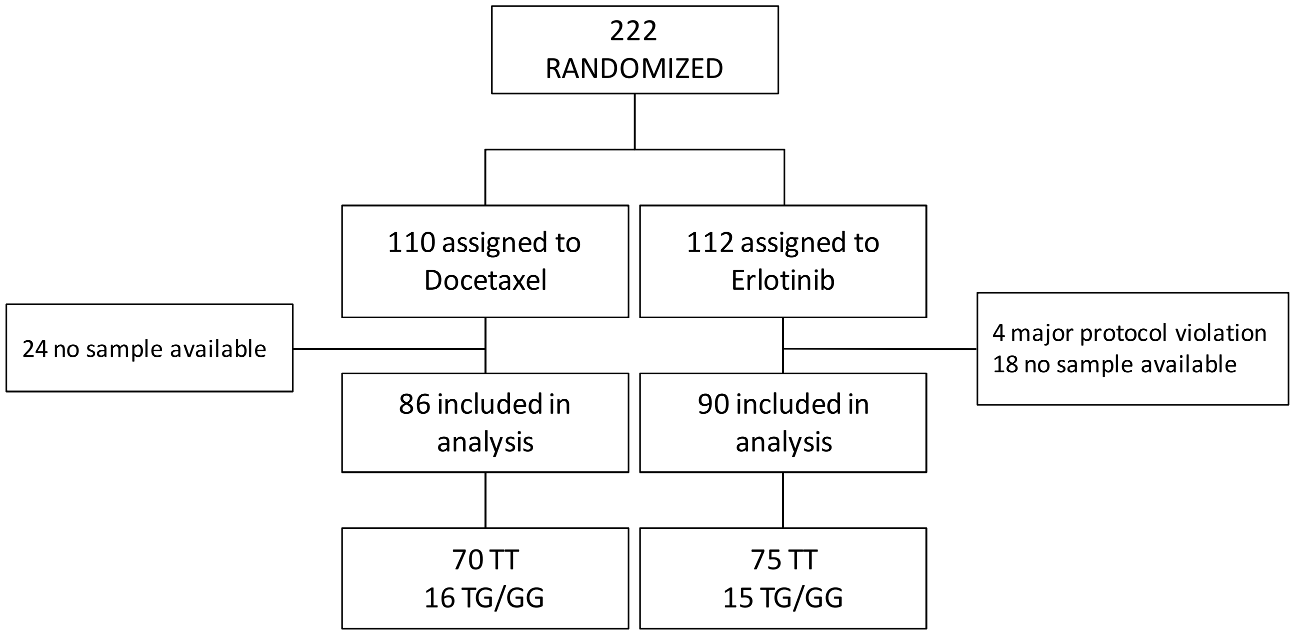

Supplementary Figure S2: Kaplan-Meier curves reporting OS (upper panels) and PFS (lower panels) in docetaxel (panels A and B) and erlotinib (panels C and D) arm according to genotypes.

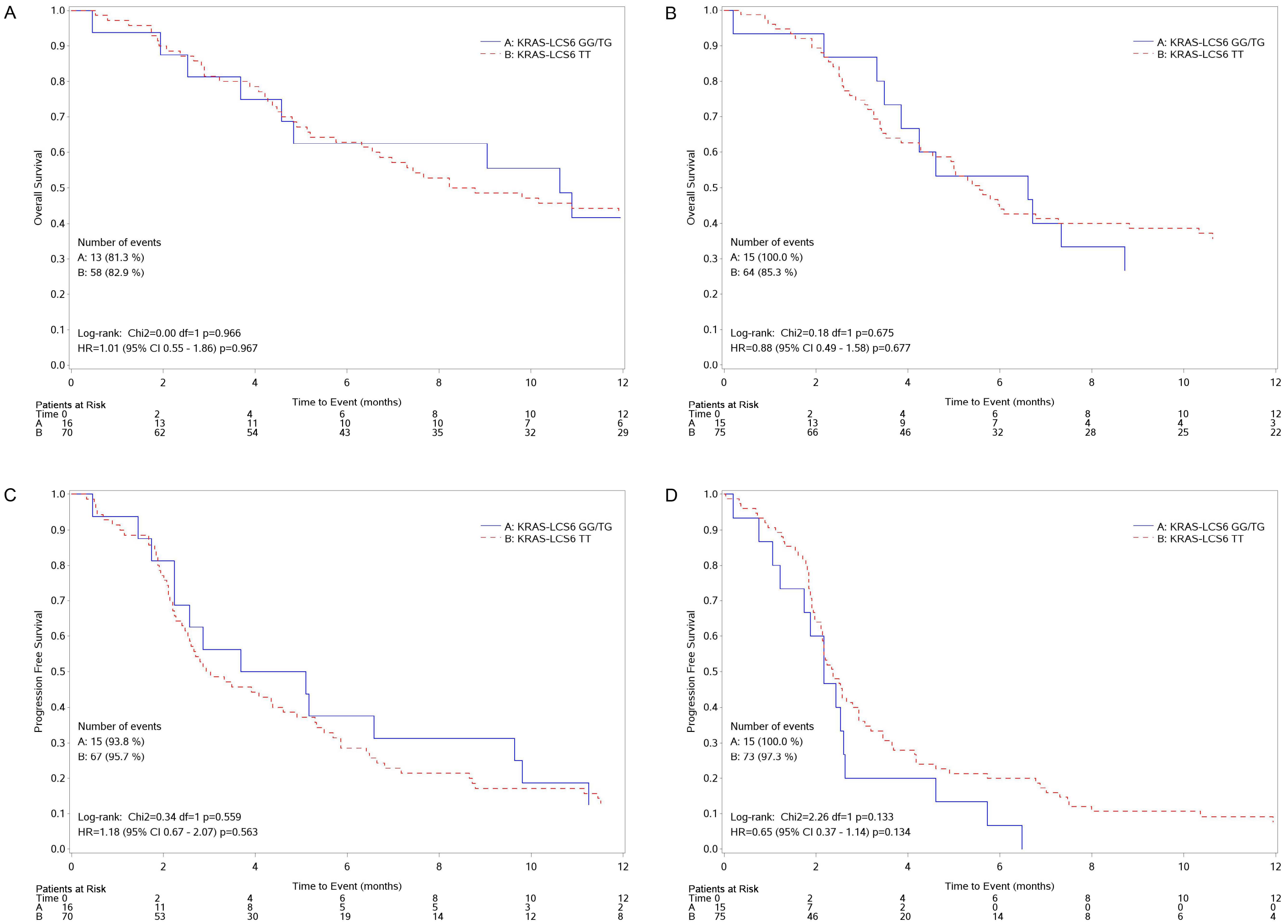

Supplement: Supplementary Information [file srep16331-s1.pdf]
